# Author Correction: PRL3 induces polyploid giant cancer cells eliminated by PRL3-zumab to reduce tumor relapse

**DOI:** 10.1038/s42003-021-02812-9

**Published:** 2021-11-08

**Authors:** Min Thura, Zu Ye, Abdul Qader Al-Aidaroos, Qiancheng Xiong, Jun Yi Ong, Abhishek Gupta, Jie Li, Ke Guo, Koon Hwee Ang, Qi Zeng

**Affiliations:** 1 Institute of Molecular and Cell Biology, Agency for Science, Technology and Research, Singapore, Singapore; 2 MD Anderson Cancer Centre, The University of Texas, Houston, TX USA; 3 Department of Cell Biology, Yale School of Medicine, New Haven, CT USA; 4 Genome Institute of Singapore, Agency for Science, Technology and Research, Singapore, Singapore; 5 Department of Biochemistry, Yong Loo Lin School of Medicine, National University of Singapore, Singapore, Singapore

**Keywords:** Cancer stem cells, Oncogenes, Cancer immunotherapy

Correction to: *Communications Biology* 10.1038/s42003-021-02449-8, published online 29 July 2021.

In the original version of the Article, the title contained an error and incorrectly spelt “polyploid” as “polypoid”. This has now been corrected in the PDF and HTML versions of the Article.

