# Supplementary material for: PRL3 induces polyploid giant cancer cells eliminated by PRL3-zumab to reduce tumor relapse
Source: Commun Biol. 2021 Jul 29;4:923. doi: 10.1038/s42003-021-02449-8 (PMC8322210; doi:10.1038/s42003-021-02449-8)
Supplement: Supplementary file 2 — Supplementary Information [file 42003_2021_2449_MOESM2_ESM.pdf]

Supplementary Information

PRL3 induces Polypoid Giant Cancer Cells eliminated by PRL3-zumab to reduce tumor relapse

Thura et al

a

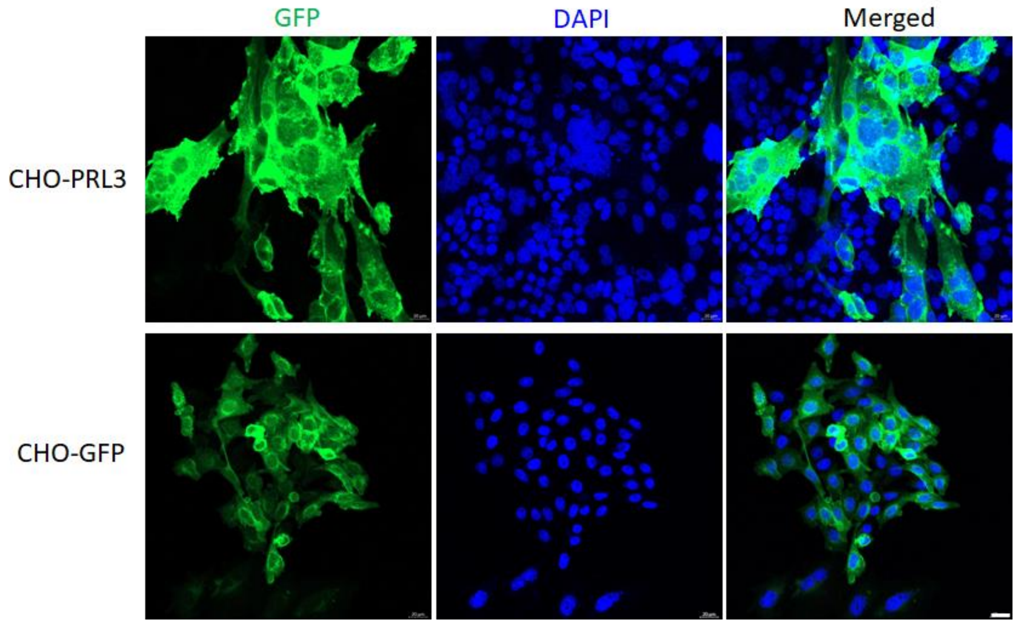

b

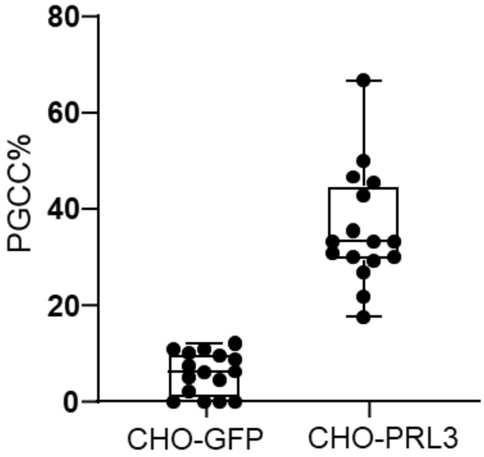

Supplementary Figure 1. PRL3 overexpression causes the formation of polyploid giant cells. (**a**) Immunofluorescence analysis of CHO cell lines stably expressing EGFP-tagged PRL3 (CHO-PRL3) and EGFP-PRL3-C104S mutant (CHO-GFP). Multinucleated polypoid giant cells can be seen abundantly in CHO-PRL3 cells. *Bar*, 20 µm (**b**) Quantitative analysis of Polypoid giant cells in CHO-GFP vs CHO-PRL3. % of PGCC was significantly higher in CHO-PRL3 cells (6.7 ± 4% in CHO-GFP vs 35.8±12% in CHO-PRL3). Data obtained from cell counts of 3 biological replicate (5-7 fields in each duplicate). P-value < 0.0001 (Students' *t*-test).

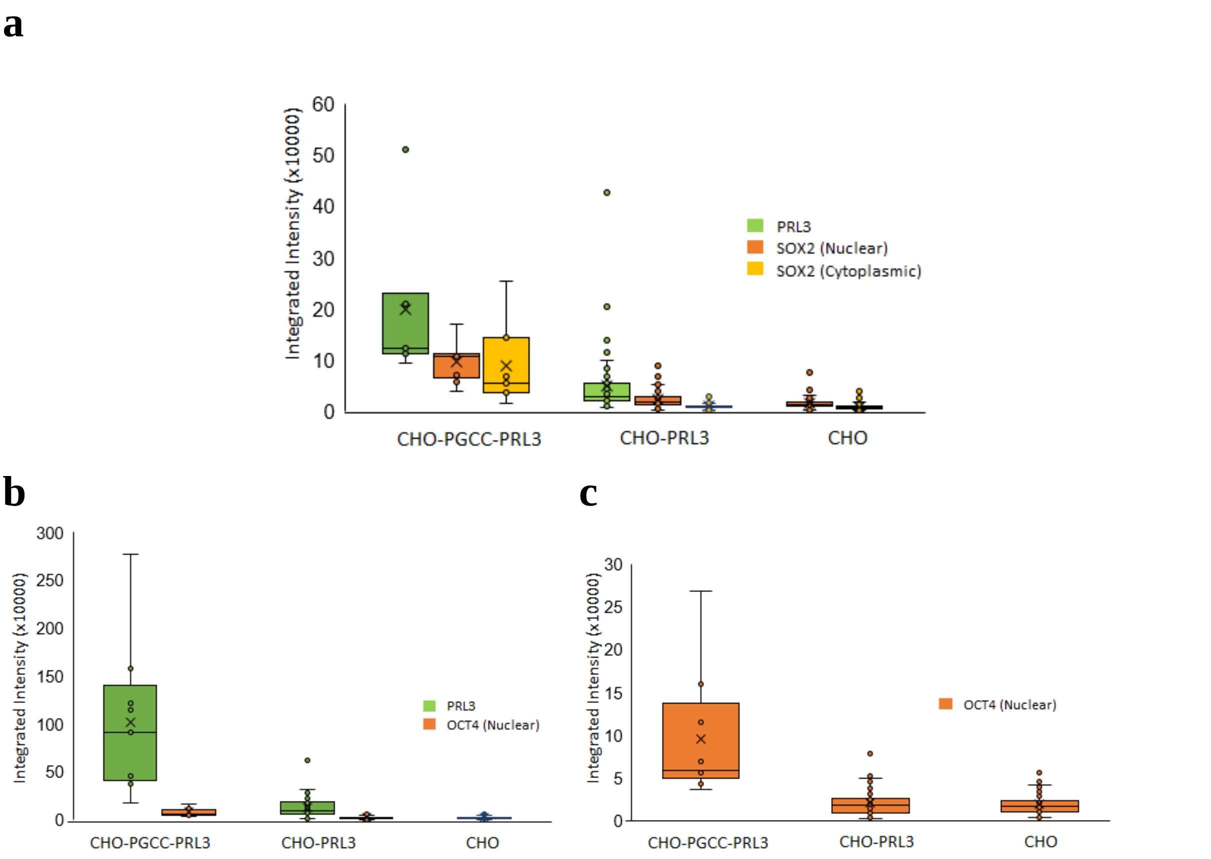

Supplementary Figure 2. SOX2 and OCT4 are highly expressed in CHO-PRL3- PGCC cells

(a) Fluorescence Intensity of PRL3 and SOX2 was analyzed in nuclear and cytoplasmic area of CHO-PRL3-PGCC cells, CHO-PRL3 cells and CHO cells of same images. Both PRL3 & SOX2 expression are significantly highest in PGCC cells. SOX2 is higher in nuclear area of all 3 cell types. P<0.0001 (Student's *t* test) for PRL3 expression. P<0.0001 (One-way ANOVA) for SOX2 expression. (b, c) Fluorescence Intensity of PRL3 (cytoplasmic) and OCT4 (nuclear) were analyzed in CHO-PRL3-PGCC cells, CHO-PRL3 cells and CHO cells of same images. (b) Both PRL3 & OCT4 expression were highest in CHO-PRL3-PGCC cells indicating High PRL3 expression is associated with high OCT4 expression. (c) Fluorescence intensity of OCT4 from (b). P<0.0001 (Student's *t*-test) for PRL3 expression. P<0.0001 (One-way ANOVA) for OCT4 expression. Data represent mean ± SD from 2 biological replicates.

a b

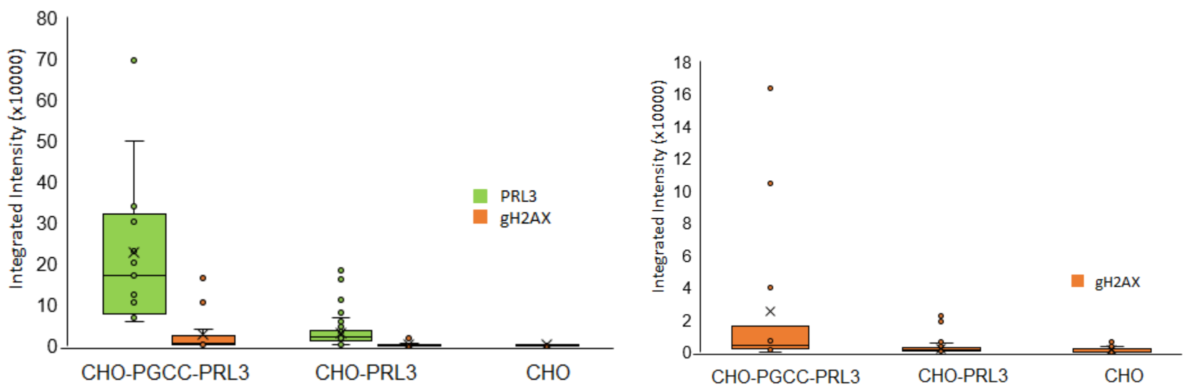

Supplementary Figure 3. γH2AX is highly expressed in CHO-PRL3- PGCC cells. Fluorescence Intensity of PRL3 (cytoplasmic) and γH2AX (nuclear) were analyzed in CHO-PRL3-PGCC cells, CHO-PRL3 cells and CHO cells of same images. (a) Both PRL3 & γH2AX expression were highest in CHO-PRL3-PGCC cells indicating high PRL3 expression is associated with high γH2AX expression. (b) Fluorescence intensity of γ γH2AX from (a). $P<0.0001$ (Student's *t*-test) for PRL3 expression. $P<0.0001$ (One-way ANOVA) for γH2AX expression. Data represent mean ± SD from 3 biological replicates.

a

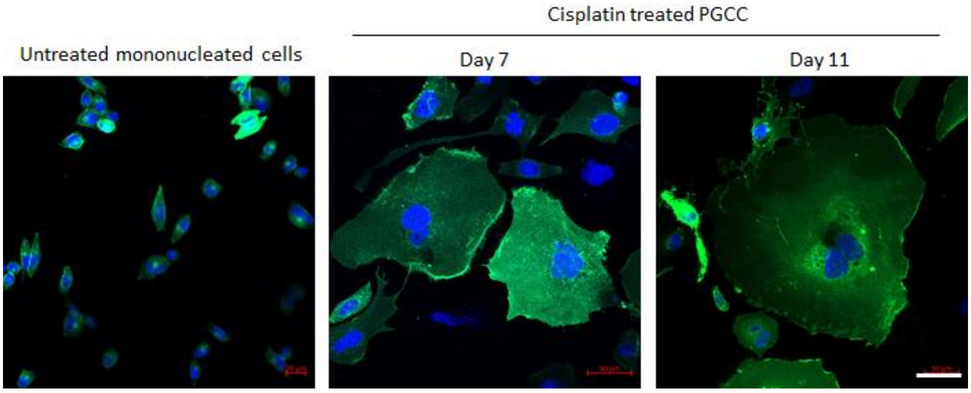

b

	Mean Area (µm²) of CHO-PRL3 cells		
	Untreated	Cisplatin treated PGCC	
		Day 7	Day 11
Mean	312.90	29676.27	31943.27
S.D	115.01	11751.79	22277.59

c

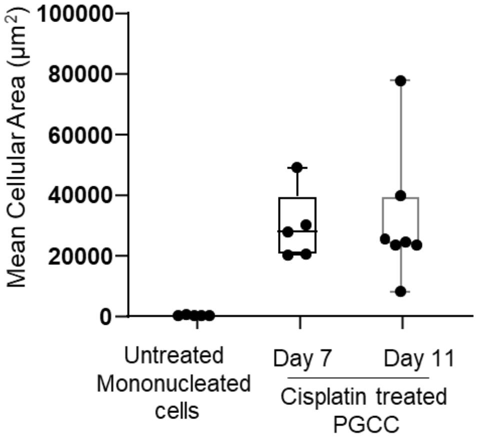

Supplementary Figure 4. Cellular area of CHO-PRL3 cells after cisplatin treatment. (a) Immunofluorescence image of CHO-PRL3 mononuclear cells at Day 0 without any treatment and PGCCs of Day 7 and Day 11 after cisplatin treatment in normal culture condition, *Bar* 50µM. (b) Mean cellular area with standard deviation for mononucleated CHO-PRL3 cells at Day 0 and cisplatin treated Day 7 & Day 11 CHO-PGCC-PRL3 cells. (c) Graph showing the data from (b). Cisplatin treated PGCC cells grew up to 100 times larger than untreated mononucleated cells. 5-7 cells from each group was randomly selected and measured for area. P=0.0001 (Untreated vs Day 7) and P=0.01 (Untreated vs Day11) (Student's *t*-test).

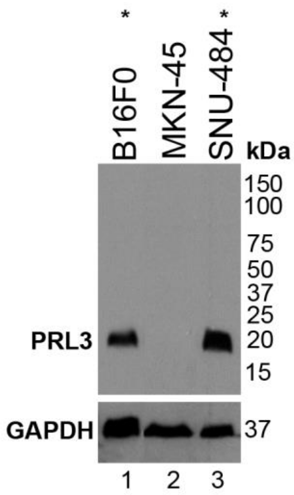

Supplementary Figure 5. Full Western blot of endogenous PRL3 in cancer cell lines.

Lane 1: B16F0 PRL3 positive mouse melanoma cell line; Lane 2: MKN-45 PRL3 negative human gastric cancer cell line; Lane 3: SNU-484 PRL3 positive human gastric cancer cell line. Glyceraldehyde 3-phosphate dehydrogenase) (GAPDH) was used as a loading control.

* PRL3 positive cancer cell lines (B16F0, SNU484) were used for 'tumor relapse model' in this study.

a

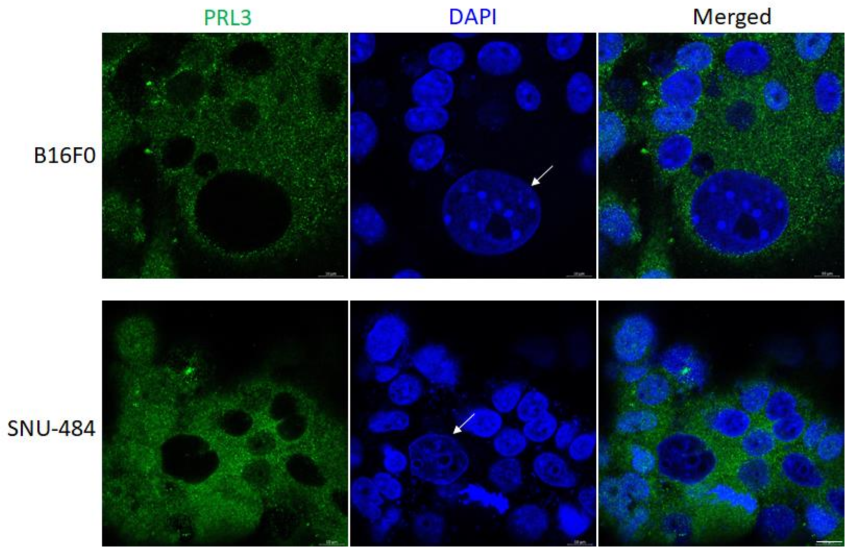

b

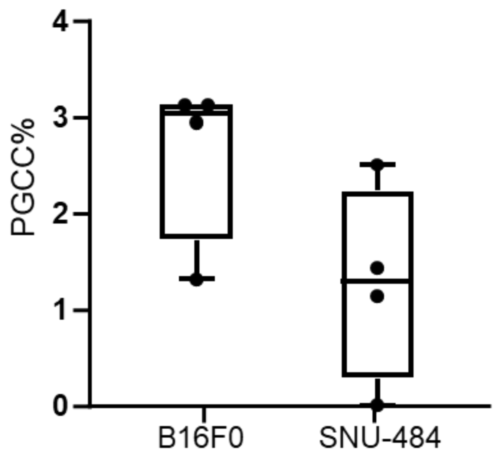

Supplementary Fig. 6. % of PGCC in B16F0 and SNU-484 cell culture

(a) Immunofluorescence images of B16F0 and SNU484 cells culture in normal condition. Green color represents PRL3. White arrow indicates PGCC. *Bar* 10μM. (b) Quantitative analysis of Polypoid giant cells in B16F0 and SNU-484 cells. % of PGCC was 2.6 ± 0.8% in B16F0 cells and 1.27±1.03% in SNU-484 cells. Data obtained from cell counts of 3 biological replicates (3 fields in each replicate).

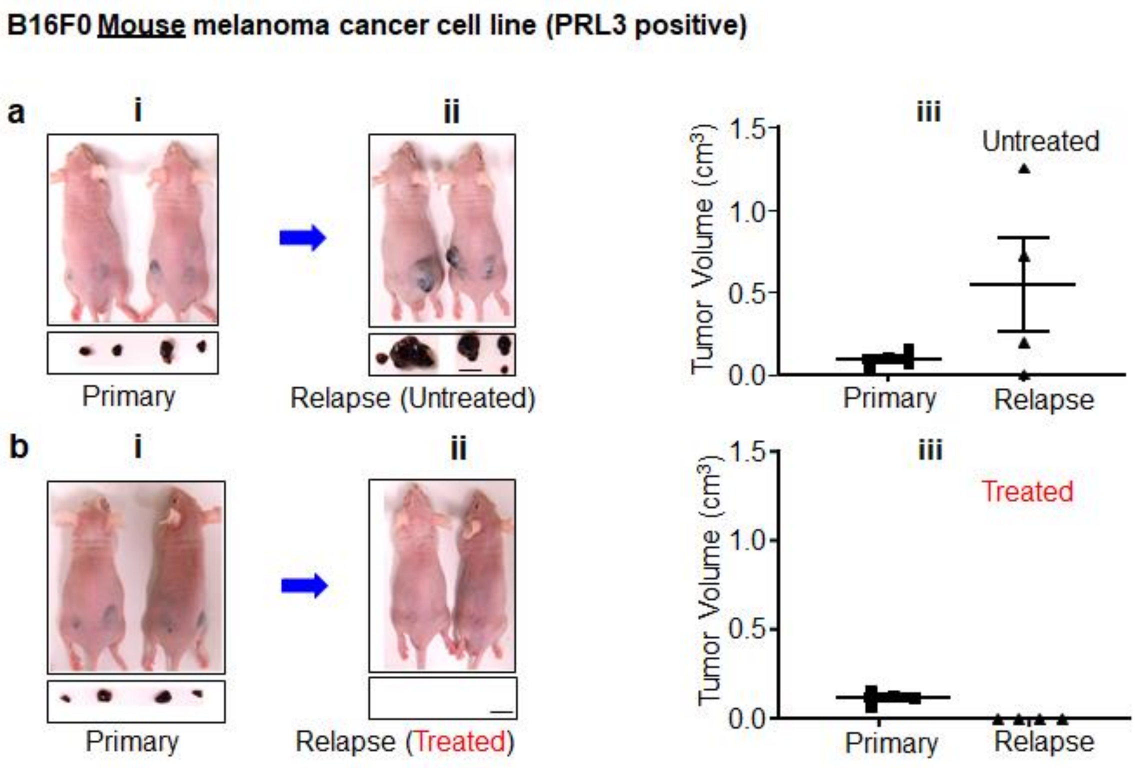

Supplementary Fig. 7. PRL3-zumab prevents PRL3 positive B16F0 tumor relapse. (a, b) Mice with xenograft tumors formed by B16F0 cells**: (a)** Mice with primary xenograft tumors at day 14 after inoculation of B16F0 cells (**a-i**, upper panel) and removed primary tumors (**a-i** lower panel). Mice (**a-i**) were kept untreated for 2 weeks after tumor removal (**a-ii**, upper panel) and removed tumors (**a-ii**, lower panel). Tumor volume of B16F0 primary and relapse tumors from a-i & a-ii (**a-iii**), n=4, data represents mean ± SEM. p= 0.19 Student's *t*-test. *Bar, 10mm*. (**b**) Mice with primary xenograft tumors at Day 14 after inoculation with B16F0 cells (b-i, upper panel) and removed primary tumors (**b-i**, lower panel). b-i mice were treated with PRL3-zumab for 2 weeks after tumor removal (**b-ii**, upper panel) and removed tumors (**b-ii**, lower panel). Tumor volume of B16F0 primary and relapse tumors from b-i & b-ii (**b-iii**), n=4, data represents mean ± SEM. P=0.009 Student's *t*-test. Tumors are appeared as block color. *Bar, 10mm.*

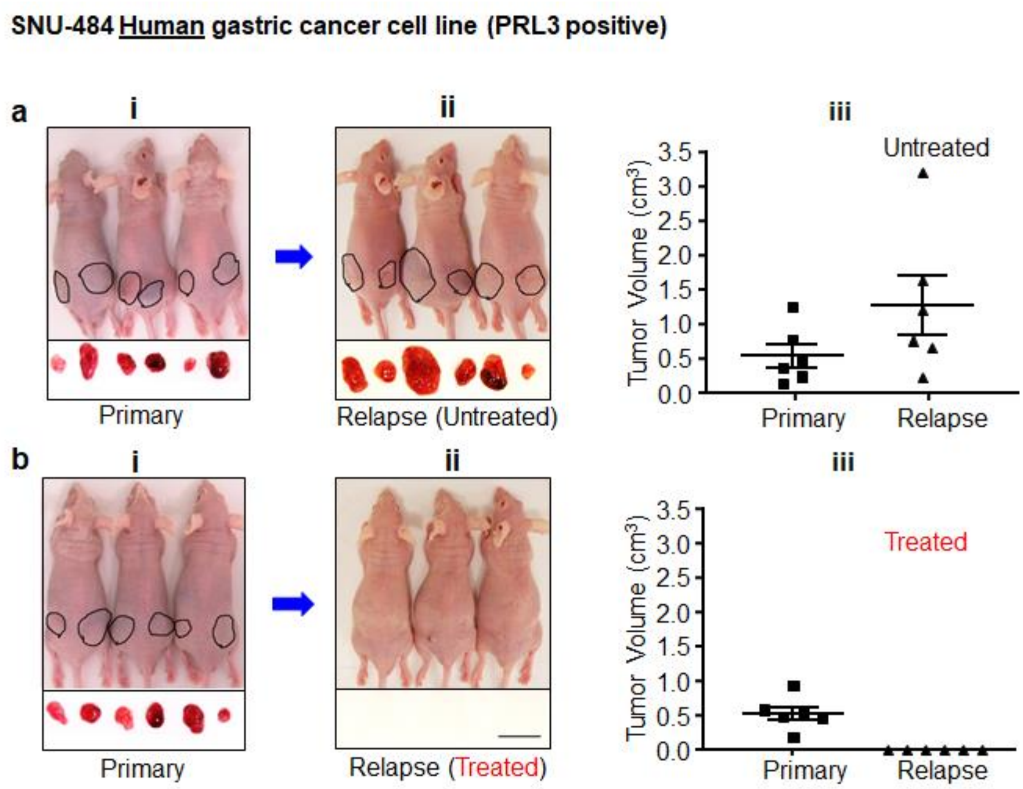

Supplementary Figure 8. PRL3-zumab prevents PRL3 positive SNU-484 tumor relapse. **(a, b)** Mice xenograft tumors formed by SNU-484 (PRL3 positive) cells: **(a)** Mice with primary xenograft tumors at day 14 after inoculation of SNU-484 cells (**a-i**, upper panel) and removed primary tumors (**a-i**, lower panel). a-i mice were kept untreated for 2 weeks after tumor removal (**a-ii**, upper panel) and removed tumors (**a-ii**, lower panel). Tumor volume of SNU-484 primary and relapse tumors from a-i & a-ii (**a-iii**), n=6, data represents mean ± SEM. p= 0.23 Student's *t*-test. *Bar, 10mm.* (**b**) Mice with primary xenograft tumors at day 14 after inoculation on SNU-484 cells (**b-i**, upper panel) and removed primary tumors (**b-i**, lower panel). b-i mice were treated with PRL3-zumab for 2 weeks after tumor removal (**b-ii**, upper panel) and removed tumors (**b-ii**, lower panel). Tumor volume of SNU-484 primary and relapse tumors from b-i & b-ii (**b-iii**), n=6, data represents mean ± SEM. P=0.003 Student's *t*-test. Black circle over mouse indicates tumor location. *Bar, 10mm.*

Fig. 2c

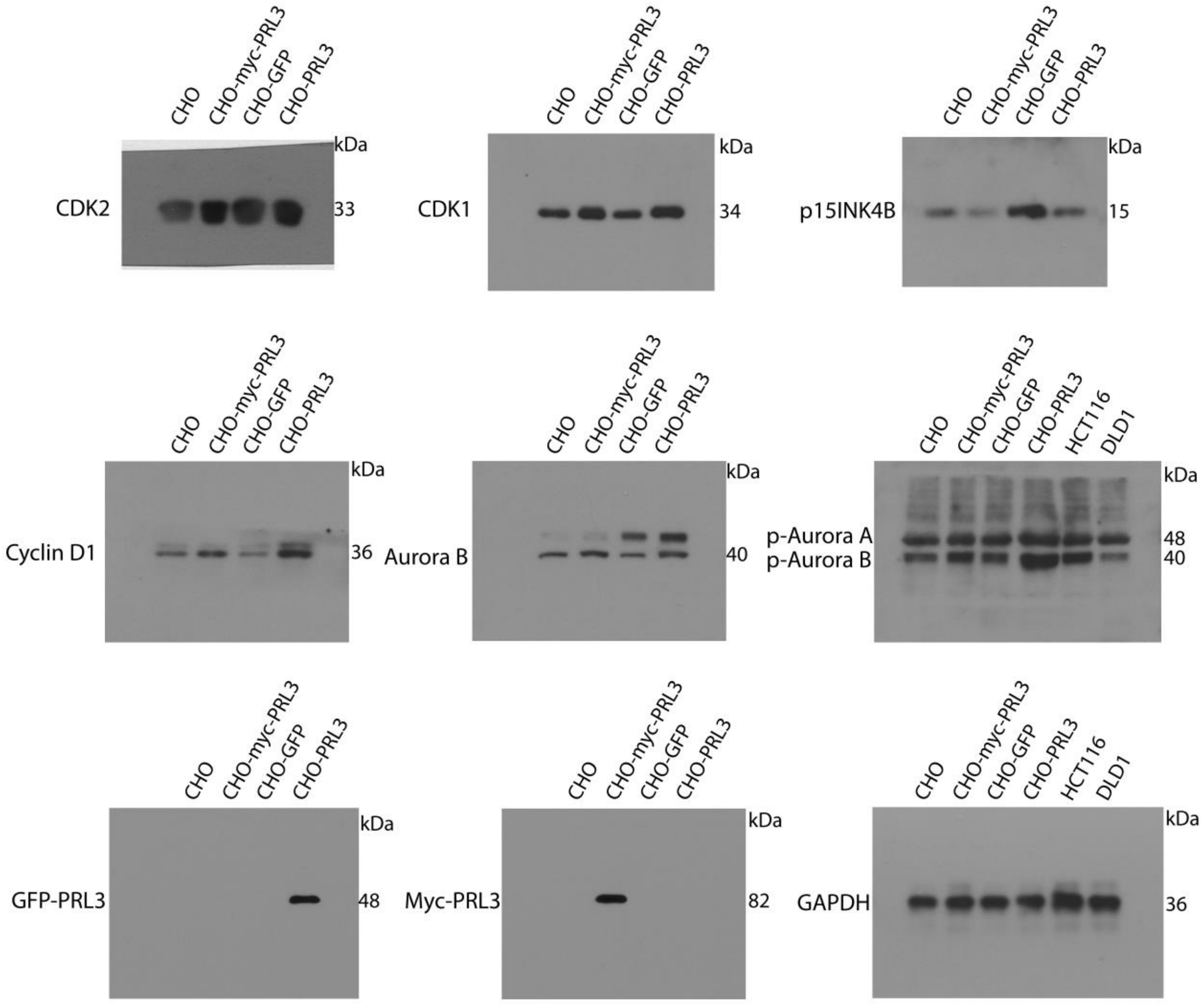

Supplementary Figure 9. Uncropped images of full western blots corresponding to displayed figure.

Fig. 2f

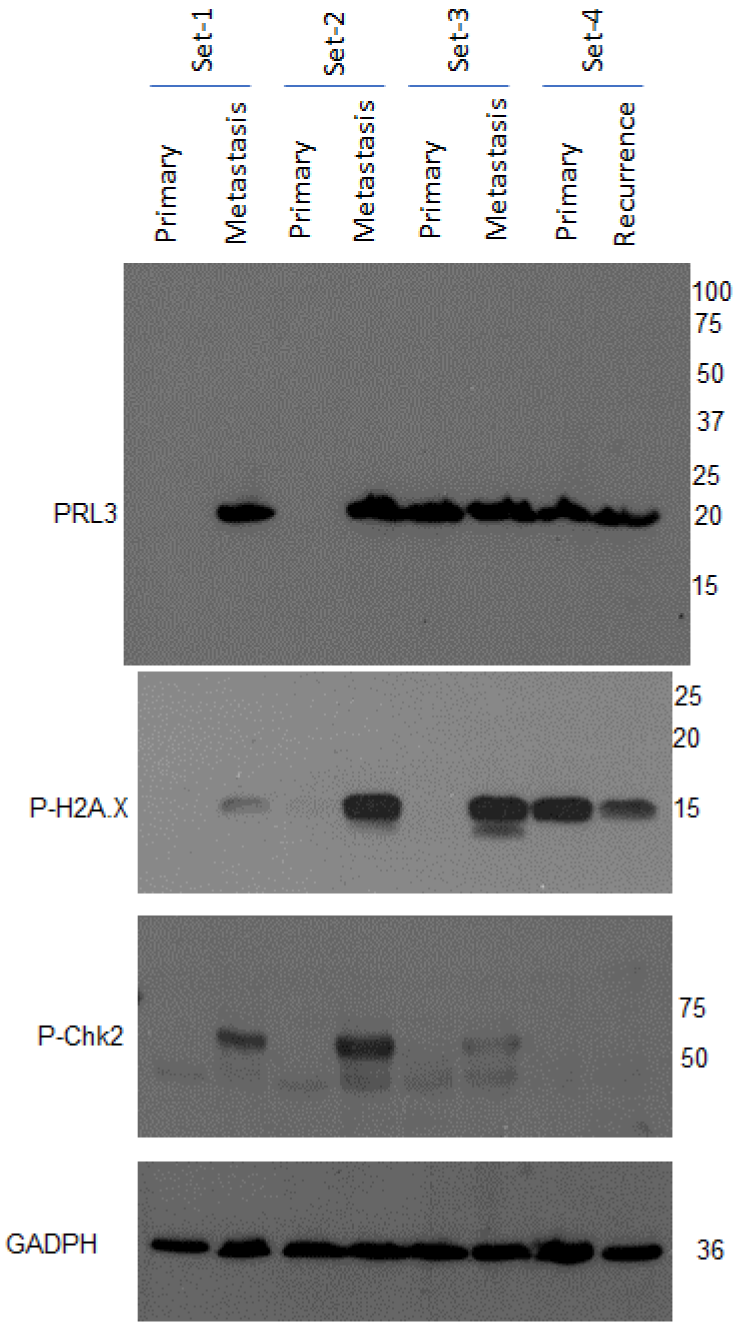

Supplementary Figure 9, *continued*

Fig. 3c

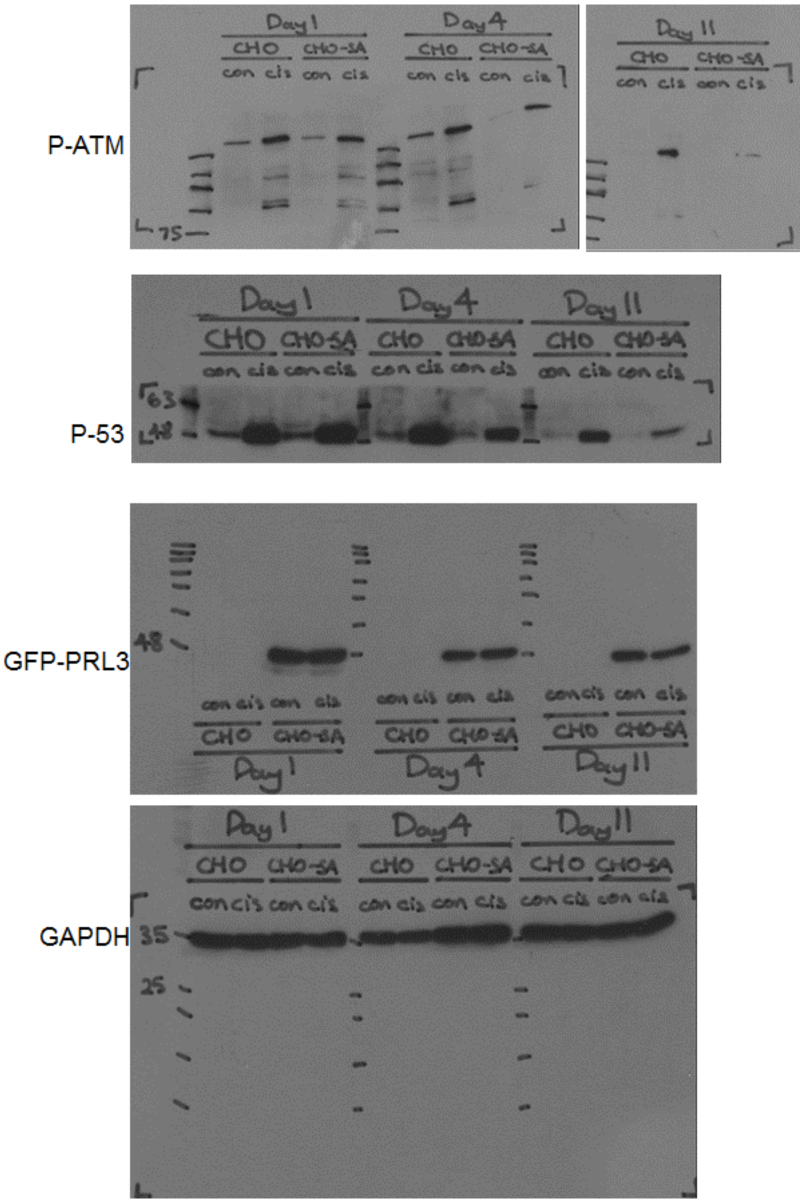

Supplementary Figure 9*, continued*

Supplementary Movie 1. PGCCs formation by incomplete cytokinesis of CHO-PRL3 cells

The movie was assembled using Zen software (Zeiss AG) from Time-lapse imaging of GFP expressing CHO-PLR3 cells in complete media scanned at 15-minute intervals for 3 days. Formation of PGCC by incomplete cytokinesis can be observed in the movie.
