# Supplementary material for: PRL3 induces polyploid giant cancer cells eliminated by PRL3-zumab to reduce tumor relapse
Source: Commun Biol. 2021 Jul 29;4:923. doi: 10.1038/s42003-021-02449-8 (PMC8322210; doi:10.1038/s42003-021-02449-8)
Supplement: Supplementary file 3 — Description of Additional Supplementary Files [file 42003_2021_2449_MOESM3_ESM.pdf]

Description of Additional Supplementary Files

File name: Supplementary Movie 1

Description: *PGCCs formation by incomplete cytokinesis of CHO-PRL3 cells*.

The movie was assembled using Zen software (Zeiss AG) from Time-lapse imaging of GFP expressing CHO-PLR3 cells in complete media scanned at 15-minute intervals for 3 days. Formation of PGCC by incomplete cytokinesis can be observed in the movie.

File name: Supplementary Data 1

Description: Source data for Figs. 2-5, Supplementary Figs. 1-4 and 6-8.
