# Supplementary material for: PRL3 induces polyploid giant cancer cells eliminated by PRL3-zumab to reduce tumor relapse
Source: Commun Biol. 2021 Jul 29;4:923. doi: 10.1038/s42003-021-02449-8 (PMC8322210; doi:10.1038/s42003-021-02449-8)
Supplement: Supplementary file 6 — Reporting summary [file 42003_2021_2449_MOESM6_ESM.pdf]

nature research

Corresponding author(s): Qi Zeng

Last updated by author(s): Jun 9, 2021

Reporting Summary

Nature Research wishes to improve the reproducibility of the work that we publish. This form provides structure for consistency and transparency in reporting. For further information on Nature Research policies, see our Editorial Policies and the Editorial Policy Checklist.

Statistics

For all statistical analyses, confirm that the following items are present in the figure legend, table legend, main text, or Methods section.

n/a	Confirmed	
☐	☒	The exact sample size (n) for each experimental group/condition, given as a discrete number and unit of measurement
☐	☒	A statement on whether measurements were taken from distinct samples or whether the same sample was measured repeatedly
☐	☒	The statistical test(s) used AND whether they are one- or two-sided<br>*Only common tests should be described solely by name; describe more complex techniques in the Methods section.*
☒	☐	A description of all covariates tested
☐	☐	A description of any assumptions or corrections, such as tests of normality and adjustment for multiple comparisons
☐	☒	A full description of the statistical parameters including central tendency (e.g. means) or other basic estimates (e.g. regression coefficient) AND variation (e.g. standard deviation) or associated estimates of uncertainty (e.g. confidence intervals)
☒	☐	For null hypothesis testing, the test statistic (e.g. F, t, r) with confidence intervals, effect sizes, degrees of freedom and P value noted<br>*Give P values as exact values whenever suitable.*
☒	☐	For Bayesian analysis, information on the choice of priors and Markov chain Monte Carlo settings
☒	☐	For hierarchical and complex designs, identification of the appropriate level for tests and full reporting of outcomes
☒	☐	Estimates of effect sizes (e.g. Cohen's d, Pearson's r), indicating how they were calculated

Our web collection on statistics for biologists contains articles on many of the points above.

Software and code

Policy information about availability of computer code

Data collection: *Provide a description of all commercial, open source and custom code used to collect the data in this study, specifying the version used OR state that no software was used.*

Data analysis: *Provide a description of all commercial, open source and custom code used to analyse the data in this study, specifying the version used OR state that no software was used.*

For manuscripts utilizing custom algorithms or software that are central to the research but not yet described in published literature, software must be made available to editors and reviewers. We strongly encourage code deposition in a community repository (e.g. GitHub). See the Nature Research guidelines for submitting code & software for further information.

Data

Policy information about availability of data

All manuscripts must include a data availability statement. This statement should provide the following information, where applicable:

- Accession codes, unique identifiers, or web links for publicly available datasets
- A list of figures that have associated raw data
- A description of any restrictions on data availability

All data generated or analysed during this study are included in this published article and its supplementary information files. Source data can be obtained in Supplementary data file.

Field-specific reporting

Please select the one below that is the best fit for your research. If you are not sure, read the appropriate sections before making your selection.

☒ Life sciences ☐ Behavioural & social sciences ☐ Ecological, evolutionary & environmental sciences

For a reference copy of the document with all sections, see nature.com/documents/nr-reporting-summary-flat.pdf

Life sciences study design

All studies must disclose on these points even when the disclosure is negative.

Sample size	For human tissue samples, we analyzed all samples that were provided by our collaborators (based on availability).<br>For animal group sizes, we used power analysis to determine the minimum number of animals required for significance based on the following calculation parameters: 1) difference in mean >= 50% (estimated according to previous experiments), 2) p value < 0.05, and 3) power = 80%.
Data exclusions	No data were excluded from analyses.
Replication	Our attempts to replicate experiments were successful.
Randomization	Tumor-inoculated mice were randomly allocated into various treatment groups. Human tissue samples were randomly provided for analysis based on availability.
Blinding	Human tissue samples, we were blinded as to whether the material provided by our collaborators were from tumor or normal areas, and received this information only after analysis.

Reporting for specific materials, systems and methods

We require information from authors about some types of materials, experimental systems and methods used in many studies. Here, indicate whether each material, system or method listed is relevant to your study. If you are not sure if a list item applies to your research, read the appropriate section before selecting a response.

Materials & experimental systems

n/a	Involved in the study	
☐	☒	Antibodies
☐	☒	Eukaryotic cell lines
☒	☐	Palaeontology and archaeology
☐	☒	Animals and other organisms
☒	☐	Human research participants
☒	☐	Clinical data
☒	☐	Dual use research of concern

Methods

n/a	Involved in the study	
☒	☐	ChIP-seq
☒	☐	Flow cytometry
☒	☐	MRI-based neuroimaging

Antibodies

Antibodies used

1) SOX2 (D6D9), Cell Signalling Technology
2) OCT4 (C30A3), Cell Signalling Technology
3) γH2AX (Ser139), Cell Signalling Technology, 9718
4) CDK1, Cell Signalling Technology, 610037
5) CDK2, Cell Signalling Technology, 610037
6) p15INK4B, Cell Signalling Technology, 4822
7) Cyclin D1 (DSS6), Cell Signalling Technology, 2926
8) p-Aurora A (T288), Cell Signalling Technology, 2914
9) p-Aurora B (T232), Cell Signalling Technology, 2914
10) Aurora B, Cell Signalling Technology, 3094
11) c-Myc (9E10), Cell Signalling Technology, D84C12
12) Phospho-Chk2 (Thr68), Cell Signalling Technology, 2197
13) GAPDH (clone MAB374), Millipore, CB1001
14) GFP (clone B-2), Santa Cruz Biotechnology, sc 9996
15) HRP- goat anti-mouse secondary antibody, Jackson ImmunoResearch
16) HRP- antirabbit secondary antibody, Jackson ImmunoResearch
17) Alexa Fluor 488 goat anti-mouse antibody, Life Technologies
18) Alexa Fluor 568 goat anti rabbit antibody, Life Technologies
19) Murine Anti-PRL3 monoclonal antibody (mAb) (clone 318), in-house

20) PRL3-zumab, generated by Wuxi Biologics
21) Phospho-ATM (Ser1981) (D6H9), Cell Signalling, 5883
22) Phospho-p53 (Ser15) 16G8mono, Cell Signalling, 9286

Validation

1) SOX2 (D6D9), Cell Signalling Technology, Validation based on manufacturer's data sheet
2) OCT4 (C30A3), Cell Signalling Technology, Validation based on manufacturer's data sheet
3) γH2AX (Ser139), Cell Signalling Technology, Validation based on manufacturer's data sheet
4) CDK1, Cell Signalling Technology, Validation based on manufacturer's data sheet
5) CDK2, Cell Signalling Technology, Validation based on manufacturer's data sheet
6) p15INK4B, Cell Signalling Technology, Validation based on manufacturer's data sheet
7) Cyclin D1 (DSS6), Cell Signalling Technology, Validation based on manufacturer's data sheet
8) p-Aurora A (T288), Cell Signalling Technology, Validation based on manufacturer's data sheet
9) p-Aurora B (T232), Cell Signalling Technology, Validation based on manufacturer's data sheet
10) Aurora B, Cell Signalling Technology, Validation based on manufacturer's data sheet
11) c-Myc (9E10), Cell Signalling Technology, Validation based on manufacturer's data sheet
12) Phospho-Chk2 (Thr68), Cell Signalling Technology, Validation based on manufacturer's data sheet
13) GAPDH (clone MAB374), Millipore, Validation based on manufacturer's data sheet
14) GFP (clone B-2), Santa Cruz Biotechnology, Validation based on manufacturer's data sheet
15) HRP- goat anti-mouse secondary antibody, Jackson ImmunoResearch, Validation based on manufacturer's data sheet
16) HRP- antirabbit secondary antibody, Jackson ImmunoResearch, Validation based on manufacturer's data sheet
17) Alexa Fluor 488 goat anti-mouse antibody, Life Technologies, Validation based on manufacturer's data sheet
18) Alexa Fluor 568 goat anti rabbit antibody, Life Technologies, Validation based on manufacturer's data sheet
19) Murine Anti-PRL3 monoclonal antibody (mAb) (clone 318), in-house, Validation Reference: Li et al, Clin.Cancer Res> 11:2195-2204 (2005)
20) PRL3-zumab, generated by Wuxi Biologics: Thura et al JCI Insight 1:e87607 (2016)
21) Phospho-ATM (Ser1981) (D6H9), Cell Signalling, 5883, Validation based on manufacturer's data sheet
22) Phospho-p53 (Ser15) 16G8mono, Cell Signalling, 9286, Validation based on manufacturer's data sheet

Eukaryotic cell lines

Policy information about cell lines

Cell line source(s)	CHO-K1 cell line (ATCC, CCL61)<br>B16F0 mouse melanoma cell line (ATCC, CRL6322)<br>SNU-484 gastric cancer cell line (KCLB)
Authentication	None of the cell lines used were authenticated.
Mycoplasma contamination	All the cell lines were tested negative for mycoplasma contamination using a PCR-based mycoplasma test kit.
Commonly misidentified lines (See ICLAC register)	None of the cell lines used are listed in the ICLAC Register (version 9).

Animals and other organisms

Policy information about studies involving animals; ARRIVE guidelines recommended for reporting animal research

Laboratory animals	NcR nude mice, males, 7-9 weeks old
Wild animals	The study did not involve wild animals.
Field-collected samples	The study did not involve the samples collected from the field.
Ethics oversight	Animal studies were approved by the A*STAR Institutional Animal Care and Use Committee (IACUC; Study No.: 161130) and performed in accordance with approved guidelines and regulations.

Note that full information on the approval of the study protocol must also be provided in the manuscript.
